# New columns in the Annals of Indian Academy of Neurology

**DOI:** 10.4103/0972-2327.70867

**Published:** 2010

**Authors:** Sanjeev V. Thomas

**Affiliations:** Editor, Annals of Indian Academy of Neurology, Department of Neurology, Sree Chithra Thirunal Institute for Medical Sciences and Technology, Thiruvananthapuram, India. E-mail: sanjeev.v.thomas@gmail.com

This issue of the AIAN carries a new column – Debates in neurology - that is set apart to discuss the controversial issues in neurology practice. Each debate would consist of a presentation of the opposing views of the subject by two experts and comments by a moderator who would put the arguments in the right perspective. The first debate would be on whether the early use of angiotensin converting enzyme (ACE) inhibitors will help in improving the outcome of stroke. We encourage the readers to participate in this debate by sending their views and comments in the form of letters to editor. We would also request the readers to suggest potential topics for future debates. This issue also contains other articles on cerebrovascular diseases and hypertension. Intracerebral hemorrhage accounts for 20% of all cerebrovascular diseases and carries a mortality of 30–50% within 1 month. One of the articles in this issue describes the outcomes of surgical management of spontaneous intracerebral hemorrhage. The approach to asymptomatic and symptomatic carotid stenosis had been in the lime light in the recent past. Several large, multicentric international trials have released their results regarding the role of carotid endarterectomy and interventional procedures. Neurologists and stroke experts have shown an increasing interest and aptitude to carry out stent procedures in carotid artery disease. These professionals are the logical first choice as patients tend to approach them first. Another article in this issue describes the experience of a medical team of neurologists in carrying out carotid stent procedures in China.

Primary writing tremor is a task-specific tremor that occurs predominantly during writing. Writer’s cramp is one of the common disorders in the movement disorder clinics. Both these disorders do not reveal any abnormalities in traditional neuroimaging. This issue of the journal carries a short communication on the functional magnetic resonance imaging (fMRI) characteristics of a primary writing tremor and writer’s cramp. They observed changes in the activation pattern of primary and secondary motor areas, cingulated gyrus and cerebellum. fMRI studies are likely to throw more light on the pathogenesis of these functional disorders.

The political history of Trichirapally (Trichy) goes back to the 3^rd^ century BC, when the early Chola ruled this part of the subcontinent from its suburbs. The city, laid out on the banks of River Cauvery, is the cradle of culture, business and education. The 18 ^th^annual meeting of the Indian Academy of Neurology will be held in this city from 24 to 26 September, 2010. The abstracts of the scientific program of this conference will be brought out by the Annals of Indian Academy of Neurology as a special supplementary issue. We wish this meeting all success.

